# The Institutional Library

**Published:** 1916-08-12

**Authors:** 


### THE INSTITUTIONAL LIBRARY.

**The After-Treatment of Operations.** By P. Lockhart Mummery, F.R.C.S. Fourth Edition. 1916. (London: Baillière, Tindall and Cox. Pp. 275. Price 5s. net.)

Mr. Mummery was, we believe, the first surgeon (in this country, at least) to write a book on the after-treatment of operations, a vastly important subject which before that had not received the full attention it deserved. That was thirteen years ago, and the high estimate which the medical profession formed of his achievement is evidenced by the issue of a fourth edition within so comparatively short a time. In this new edition a chapter on military surgery is included, and the author in his preface somewhat anticipates the criticism of scrappiness, irrelevance, and inadequacy which is sure to be levelled against it by expressing his own misgivings; apparently he has been led into this eminently topical divagation rather against his better judgment. The chapter on Shock has been rewritten: the author is well known as a strong adherent of one particular hypothesis of this condition, and makes no attempt to put the views of the many who dissent from it. The principal opponent of the school to which Mr. Mummery belongs is contemptuously dismissed in a line and a half, with two errors in the spelling of his name. Indeed, the spelling (more probably the proof-reading) is weak in other places: thus on one page we meet with "rung" for "wrung" and "linamentum" for "linimentum"; and the index is a very poor one. The author deserves well of the medical public, but he must look more closely to his laurels if he is to keep in the forefront of his rivals.

**More Minor Horrors.** By A. E. Shipley, Sc.D. (London: Smith, Elder and Co. 1916. Pp. 163. Price 1s. 6d. net.)

This extension of the very entertaining and popular account which the Master of Christ's College gave some time ago of the flea, the louse, the bug, and similar tormentors of mankind, will doubtless be as great a success as the original was, in spite of the traditional fate of all sequels. In this volume a series of animals is dealt with which annoy the sailor and the soldier in the Tropics more than do the beasts described in the former volume. The mosquito, the rat, the cockroach, the bot-fly, the stable-fly, and one or two others are catalogued in the humorous yet strictly accurate and scientific style that his readers expect of Dr. Shipley. We predict a popularity for this sequel almost, if not quite, equal to that of its predecessor.

**A Practical Book for Tuberculosis Officers.**

**I.K. Therapy in Pulmonary Tuberculosis.** By William Barr, M.D., D.Sc. Glasg., D.P.H. Camb. (Bristol: John Wright and Sons, Ltd. 1916. Price 3s. 6d. net.)

This is a really practical handbook for those who have occasion to deal with cases of phthisis. It contains simple and concise instructions for the correct administration of "I.K.," the now generally accepted abbreviation for Spengler's preparation "Immunkörper" (or immune substances), which is given in a somewhat similar manner to tuberculin. I.K. is, however, less toxic than tuberculin, and it appears to produce results rather sooner; consequently it is peculiarly adapted for dispensary treatment. Numerous tables and charts are given showing the effect of the injections upon the body weight, the temperature, and the amount of sputum. In the summary of forty-seven cases given at the end of the book the best results are, naturally, those obtained in the early cases, but in several of those in Stage III. (Turban) much benefit and power to work is reported. Altogether this book is one worthy of careful study by tuberculosis officers and others who are called upon to treat pulmonary consumption.

**Red Cross and Iron Cross.** By a Doctor in France. (London: John Murray. 1916. Pp. 142. Price 2s. 6d. net.)

The scene of this glimpse of the appalling horrors of modern war, especially of the kind of war waged by the Huns, is a small village in which the retreating French have left behind a collection of their own and of German wounded, most of them already moribund or too bad to be moved. An English—or is it American?—doctor of some undescribed Red Cross organisation is supposed to arrive very opportunely, all by himself, and to assist in the care of the dying men. Presently the German cavalry enter the village, and their officers proceed to bullyrag the mayor, the curé, the doctor, and the rest of the inhabitants, *more suo*. The whole book is filled with incidents of German brutality and is useful in that it emphasises what many people in this uninvaded island so easily forget, the immeasurable horror of German militarism and the nameless infamy of the German Army. There is, however, one very serious bone which we have to pick with the anonymous author (we doubt very strongly whether he is a Doctor in France, by the way). He endeavours to create the impression, without actually saying so in words, that these pages contain a true record of facts, not a work of imagination; and the publisher would seem to have some part of this responsibility to bear also. Between them they may mislead the British public, for it is quite evident that the story is imaginative, and in many essentials it is not even true either to life or to death.

**Under Three Flags: With the Red Cross in Belgium, France, and Serbia.** By St. Clair Livingston and Ingeborg Steen-Hansen. (London: Macmillan and Co. 3s. 6d. net.)

This book differs from most of its class in that it does not give the description of incidents in hospital as seen from the point of view of a hospital nurse, a diary of routine in travelling or in hospital wards. It gives rather the impression of being a string of incidents in various places which might have been witnessed by anyone who had happened to be travelling about the Allied countries since the war began. The English author's statement that journalism as well as nursing has been her occupation perhaps accounts for this; but if a little more space had been given to the reasons and duties which took her from place to place the whole would have read more coherently and less like a series of cinematograph pictures. On the other hand, the volume makes more lively reading than most of these war-diaries, for the author has moved about, and thrown into her pages any story that she came across, and if the whole had not been so formless it might have been not merely a publication, but a book. It is, in fact, the jottings rather of a journalist than of a nurse. Miss Steen-Hansen has written her version of their joint adventures in Norwegian. Each book, though different, is bound in the same material and arranged in the same way.

**Downward Paths.** (London: G. Bell and Sons. 1916. Pp. 200. Price 2s. 6d. net.)

We find it difficult to overpraise the spirit in which this book has been put together and the value to all social reformers of the facts it contains. It is an inquiry into the causes which contribute to the making of the prostitute in Great Britain, and it really does appear that its anonymous women authors have devoted themselves, as they claim, to the collection of accurate knowledge rather than the forwarding of any pet piece of legislation or the propping up of any pre-formed hypothesis. They had not decided beforehand what they were going to find, and they recognise that a lack of such detachment has nullified much of the most well-meaning work done by women in the last few years for the mitigation of the "Social Evil." The results of this inquiry do not pretend to be exhaustive or final; the authors recognise that their statistics are vitiated by too many disturbing factors to be applicable to this country as a whole. Nevertheless, we feel that they have got much nearer the heart of the strange problem of prostitution than many of those who have most to say on this topic, and that their valuable spade-work will, be the foundation of efforts more successful because more logical than the spasmodic and too often semi-hysterical measures in favour with many protagonists of the feminist movement. Obviously a book such as this does not lend itself to quotation; it should be read by every man and woman in the land. But we must just mention the stinging rebuke dealt out to the authors of sensationalism of the "Where are you going to?" type, whose materials are shown to be a tissue of falsehood from beginning to end.

**Surgical Nursing and Technique.** By Charles P. Childe, B.A., F.R.C.S. Second Edition. Pp. 229. (London: Baillière, Tindall and Cox. Price 3s. 6d. net.)

We have perused this book with much pleasure and profit, and we quite agree with the author that its contents will be useful to students, dressers, and junior practitioners, although the author is addressing himself particularly to nurses. At the present time we believe the book will be more especially useful, in view of the large amount of surgical work and surgical nursing entailed by the war. The use of a considerable number of technical terms, which the author has not defined, necessitates on the part of the nurse a previous acquaintance with a more elementary nursing manual. Indeed, the author is addressing himself rather to the already partially trained nurse.

In Chapter I. the most desirable qualities in a surgical nurse are described, and no nurse can fail to profit by assimilating the good advice there given. Incidentally the author refers in a sympathetic manner to the hardships, financial and otherwise, cheerfully endured by the present-day hospital sister and nurse, and urges the union and organisation necessary to demand better conditions of life and an increased recognition of their great services to the public. Chapter III. describes the systematic preparation of a patient for operation, from the time of admission to hospital (or home). On page 31 we find: "In man, as in all warm-blooded animals, the temperature of the body remains constant. . . ." This is not strictly accurate. The chapter dealing with the patient in the theatre and the duties of the sisters and nurses is very full and concise.

Next follows the management of the patient after operation, with general directions according to the presence or absence of complications and sequelæ. This chapter closes with the after-dressing of the wound. In the following chapter the selection of instruments is discussed, and the figures illustrating many of the commoner instruments in use are helpful. The book closes with two short chapters dealing with operations in surgical homes and private houses and nursing in military hospitals.

**The Campaign for Clean Milk.** (London: The St. Catharine Press, for the National Clean Milk Society. Price 1s. net.)

This pamphlet is a reprint of a series of articles which appeared a few months ago in the *Observer*. The articles do great credit to the society under whose auspices they were prepared, and it is a very gratifying sign of the times that a paper so widely read and so influential as the *Observer* was willing to print them. The case here disclosed is not overstated; there is neither exaggeration nor sensation-mongering. It reveals a state of affairs which must be altered; and, although the war almost monopolises public interest at the moment, we believe the public will see that it shall be altered. Only knowledge of the facts is required, and that will be best disseminated by publications such as this, which deserve the widest of circulations.

**A Text-Book of Physics and Chemistry for Nurses.** By A. R. Bliss, M.D., and A. H. Olive, Ph.Ch. (London and Philadelphia: J. B. Lippincott Company. Pp. 240. 6s. net.)

While the title states that this book is for nurses, the preface amplifies it by adding that it is intended for the student and "graduate" nurse.

The scope of the book may be indicated thus: Part I. deals with elementary physics; Parts II. and III. with organic and inorganic chemistry; Parts IV. and V. with physiological chemistry, fermentation, and ferments.

It will be seen that the ground covered is both varied and comprehensive, while the method of treatment is quite out of the usual run of text-books dealing with these subjects. The style throughout is interesting, the text is clear, and the illustrations are numerous. The bearing of each subject is largely pharmaceutical, and the therapeutic aspect is kept prominently in view. The section on urine analysis is excellently done, and the same remark holds good for that on disinfectants and antiseptics. The appendix includes a chapter on metric equivalents, and a most complete and useful glossary. The importance of the book lies in its use as a text-book covering all the subjects mentioned, and the nurse will be able to assimilate the teaching even if she has not previously qualified in physics and chemistry. An excellent index concludes a book which cannot fail to prove a valuable addition to the nurse's educational equipment.

**Surgical Bandages and Dressings.** Abridged Edition. Diagrams by Alice Scott. (London: Percy Lund, Humphries, and Co., Ltd. 1s. net.)

This little book contains a fairly complete collection of bandages, and the patterns are good ones. The diagrams, however, seem to us incomplete, as they are unaccompanied by paper patterns; and few war workers could cut out a shoulder, hip, or capelline bandage without a pattern. The arm-sling which crosses at the back, and so prevents any weight on the patient's neck, seems a good one, while the Robertson Lawson head and neck bandage would be invaluable for certain cases. The dressings, too, are inadequately described, and it is not likely that this book would be of use to war workers unacquainted with surgical needs, though it might help to remind those who had been shown what was needed when memory failed.

